# Supplementary material for: Involvement of circulating factors in the transmission of paternal experiences through the germline
Source: EMBO J. 2020 Oct 9;39(23):e104579. doi: 10.15252/embj.2020104579 (PMC7705452; doi:10.15252/embj.2020104579)
Supplement: Supplementary file 1 — Appendix [file EMBJ-39-e104579-s001.pdf]

Appendix

Authors: Gretchen van Steenwyk[1,2,3†], Katharina Gapp[2,3,4,5,6,7†], Ali Jawaid[1,2,3,10], Pierre-Luc Germain[1],[2,8], Francesca Manuella[1,2,3], Deepak K. Tanwar[1,2,3,8], Nicola Zamboni[9], Niharika Gaur[1,2,3], Anastasiia Efimova[1,2,3], Kristina M. Thumfart[1,2,3], Eric A. Miska[5,6,7], and Isabelle M. Mansuy[1,2,3*]

Affiliations:[1]Laboratory of Neuroepigenetics, Medical Faculty of the University of Zurich, Brain Research Institute, Winterthurerstrasse 190, Zurich, Switzerland.

[2]Institute for Neuroscience, Department of Health Sciences and Technology, ETH Zurich, Zurich, Switzerland.

[3]Zurich Neuroscience Center, ETH Zurich and University of Zurich, Zurich, Switzerland.

[4]Laboratory of Molecular and Behavioral Neuroscience, ETH Zurich, Winterthurerstrasse 190, Zurich, Switzerland.

[5]Gurdon Institute, University of Cambridge, Tennis Court Rd, Cambridge, CB2 1QN, UK.

[6]Wellcome Trust Sanger Institute, Hinxton, UK.

[7]Department of Genetics, University of Cambridge, Downing Street, Cambridge, UK.

[8]Statistical Bioinformatics Group, Swiss Institute of Bioinformatics, Zurich, Switzerland.

[9]Institute of Molecular Systems Biology, ETH Zurich, Zurich, Switzerland.

[10]Laboratory of Translational Research in Neuropsychiatric Disorders, BRAINCITY Nencki-EMBL Center of Excellence for Neural Plasticity and Brain Disorders, Warsaw, Poland.

*Correspondence to: mansuy@hifo.uzh.ch

†Authors share equal contribution

This PDF file includes:

Appendix Figures S1-23

Appendix Figures

Enrichment	MSUS adults		MSUS offspring	
	-	+	-	+
Alpha linolenic acid and linoleic acid metabolism	/	5.2E-05	5.0E-03	2.1E-02
Arachidonic acid metabolism	/	3.5E-05	/	/
Arginine and proline metabolism	2.9E-02	/	/	/
Bile acid biosynthesis	1.9E-09	/	1.4E-14	/
Galactose metabolism	/	/	/	5.1E-05
Pentose phosphate pathway	2.1E-02	/	/	/
Retinol metabolism	/	/	/	/
Steroidogenesis	8.8E-02	/	1.2E-02	/

Appendix Figure S1

Table expanded from Fig. 1C to show all metabolomic enrichment pathways in MSUS males with a false discovery rate (FDR) < 0.1 (to show all significant enrichments as well as trends) after multiple testing corrections using the Benjamini-Hochberg (BH) test. (+) denotes a positive enrichment, (-) denotes a negative enrichment. FDR reported with the scientific numbering system. (/) symbolizes non-significance.

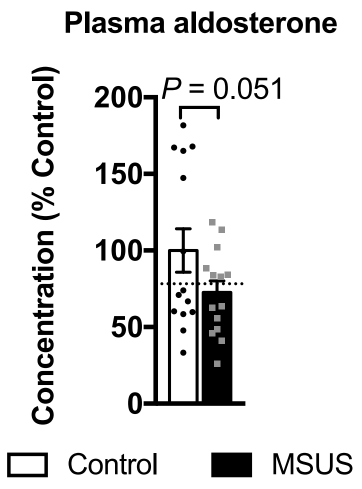

Appendix Figure S2

Concentration of aldosterone in plasma from MSUS and control males measured by ELISA. The dotted line at 78.2% indicates fold change observed by previous TOF-MS measurement (FDR = 0.02). One-tailed Student's *t*-test, $n = 14$ per group, $t = 1.70$, df = 26. FDR; false discovery rate. Data reported as mean ± s.e.m.

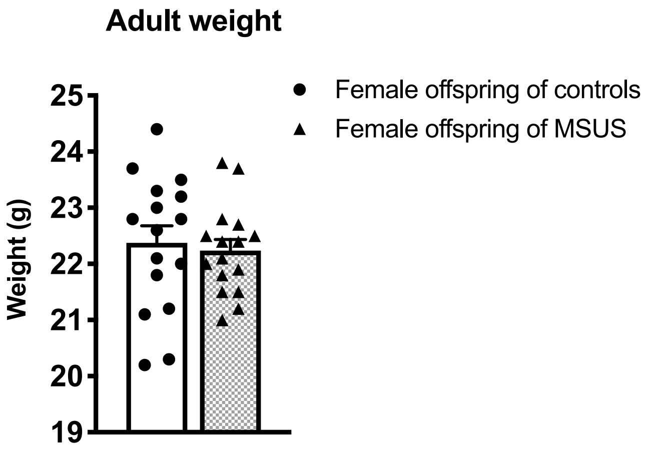

Appendix Figure S3

Bodyweight in adult female offspring of MSUS males.

Mean weight was comparable between MSUS and control female offspring of MSUS males (Control n = 12, MSUS n = 12), $t(39) = 0.38, P > 0.05)$.

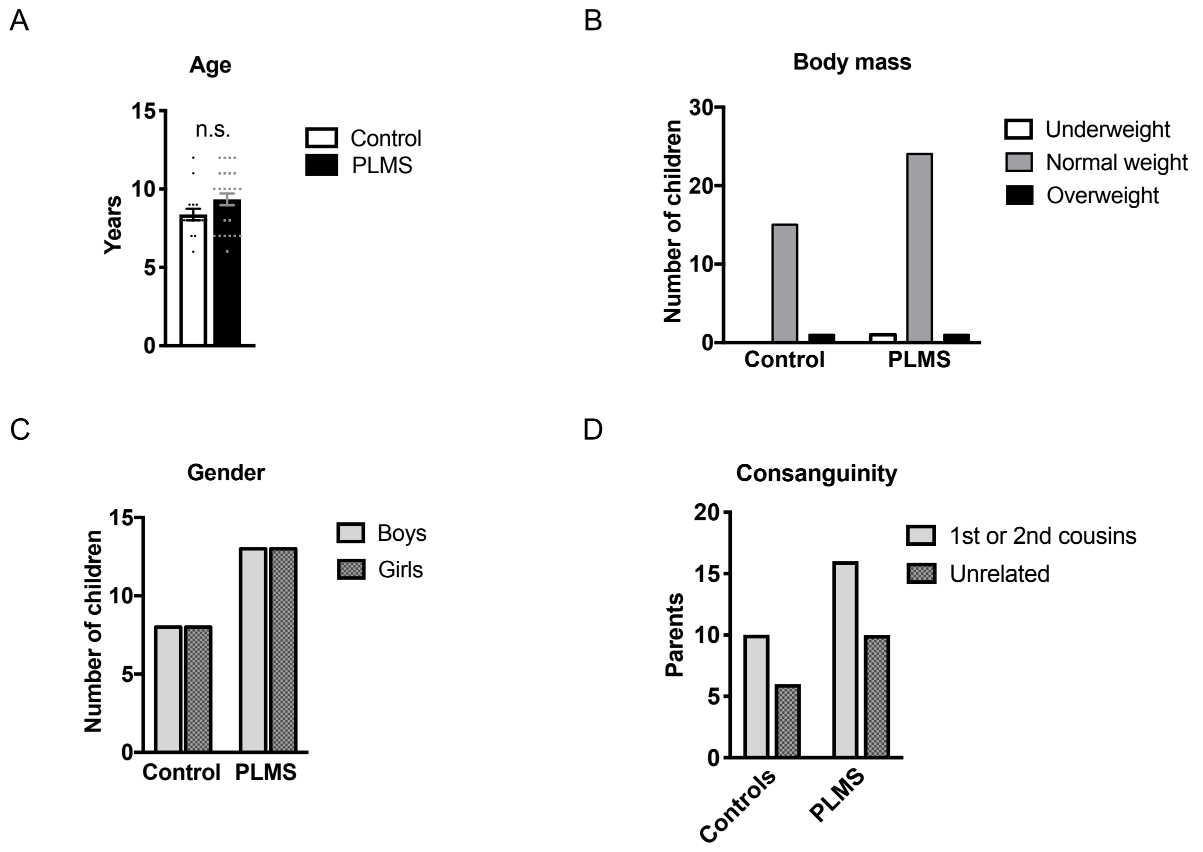

Appendix Figure S4

Descriptive data for children in PLMS and control groups.

(A) Mean age was comparable between groups. PLMS *n* = 26, Control *n* = 16, two-tailed Mann-Whitney *U* = 148, *P* = 0.12. n.s.; not significant. (B) Body mass in children was classified as underweight, normal weight or overweight based on correspondence to reference ranges defined for Pakistani children of same age and sex. In both groups, one child was overweight while in the PLMS group, one child was underweight. All other children were within the range of normal body mass. (C) Each group has an equal number of boys and girls to balance gender-specific effects. PLMS boys *n* = 13, girls *n* = 13; Control boys *n* = 8, girls *n* = 8. (D) Number of control and PLMS children with consanguinity in parents defined by 2nd or 3rd cousins. PLMS *n* = 26, Control *n* = 16. Data reported as mean ± s.e.m., n.s., not significant.

Enrichment in PLMS children	Serum		Saliva	
	-	+	-	+
Alanine Metabolism	/	8.7E-04	/	/
Alpha Linolenic Acid and Linoleic Acid Metabolism	/	/	/	6.1E-04
Amino Sugar Metabolism	8.0E-02	1.7E-04	/	/
Ammonia Recycling	/	2.5E-05	/	/
Androgen and Estrogen Metabolism	/	2.6E-02	/	/
Arachidonic Acid Metabolism	/	6.1E-28	/	6.3E-16
Arginine and Proline Metabolism	/	4.6E-07	4.7E-04	/
Aspartate Metabolism	/	6.9E-04	/	/
Beta Oxidation of Very Long Chain Fatty Acids	/	4.3E-02	/	/
Beta-Alanine Metabolism	/	6.3E-03	/	/
Betaine Metabolism	/	2.9E-02	/	/
Butyrate Metabolism	/	/	2.6E-02	/
Carnitine Synthesis	/	1.0E-03	/	/
Catecholamine Biosynthesis	/	/	2.1E-02	/
Citric Acid Cycle	/	3.6E-03	6.9E-06	/
Cysteine Metabolism	/	8.7E-04	/	/
Fatty Acid Biosynthesis	/	9.4E-02	2.0E-02	/
Folate Metabolism	/	8.0E-03	/	/
Fructose and Mannose Degradation	2.4E-05	/	6.2E-03	/
Galactose Metabolism	2.8E-11	/	9.0E-13	/
Gluconeogenesis	5.7E-05	6.3E-03	1.8E-05	/
Glucose-Alanine Cycle	3.2E-02	4.8E-03	/	/
Glutamate Metabolism	/	3.3E-03	2.6E-02	/
Glutathione Metabolism	/	3.8E-04	/	/
Glycerol Phosphate Shuttle	/	2.7E-02	/	/
Glycerolipid Metabolism	/	4.5E-03	2.8E-02	/
Glycine and Serine Metabolism	6.8E-02	1.1E-06	3.4E-02	/
Glycolysis	4.4E-05	8.5E-02	4.7E-04	/
Histidine Metabolism	/	1.7E-02	/	/
Homocysteine Degradation	/	/	4.4E-02	/
Inositol Metabolism	4.0E-02	4.6E-02	4.0E-02	/
Inositol Phosphate Metabolism	4.5E-02	6.9E-02	/	/
Lactose Degradation	5.7E-05	/	6.7E-04	/
Lactose Synthesis	3.4E-02	/	1.2E-03	/
Malate-Aspartate Shuttle	/	4.3E-04	/	/
Methionine Metabolism	/	3.6E-03	/	/
Mitochondrial Electron Transport Chain	/	4.8E-03	1.2E-02	/
Nucleotide Sugars Metabolism	7.1E-04	/	4.7E-04	/
Phenylalanine and Tyrosine Metabolism	/	7.0E-02	5.8E-03	/
Phosphatidylinositol Phosphate Metabolism	3.2E-02	/	/	/
Phospholipid Biosynthesis	/	8.0E-03	/	/
Plasmalogen Synthesis	/	4.5E-03	/	/
Porphyrin Metabolism	/	8.0E-03	/	/
Propanoate Metabolism	/	6.3E-03	4.7E-04	/
Pyrimidine Metabolism	/	1.3E-05	/	/
Pyruvaldehyde Degradation	/	1.0E-02	8.0E-03	/
Pyruvate Metabolism	/	6.0E-05	4.0E-04	/
Retinol Metabolism	4.0E-02	/	/	/
Sphingolipid Metabolism	4.9E-02	/	/	/
Starch and Sucrose Metabolism	1.9E-03	8.2E-02	4.7E-04	/
Steroidogenesis	/	/	/	2.5E-03
Taurine and Hypotaurine Metabolism	/	4.9E-04	/	/
Transcription/Translation	/	2.0E-05	/	/
Transfer of Acetyl Groups into Mitochondria	3.2E-02	2.8E-02	2.8E-03	/
Trehalose Degradation	3.4E-02	/	2.0E-02	/
Tyrosine Metabolism	/	9.1E-02	1.6E-05	/
Urea Cycle	5.2E-02	1.5E-03	/	/
Valine, Leucine and Isoleucine Degradation	/	2.0E-02	4.3E-04	/

Appendix Figure S5

Table expanded from Fig. 1C to show all metabolomic enrichment pathways in PLMS samples with FDR < 0.1 (to show all significant enrichments as well as trends) after multiple testing corrections using the Benjamini-Hochberg (BH) test. (+) denotes a positive enrichment, (-) denotes a negative enrichment. FDR reported with the scientific numbering system. (/) symbolizes non-significance.

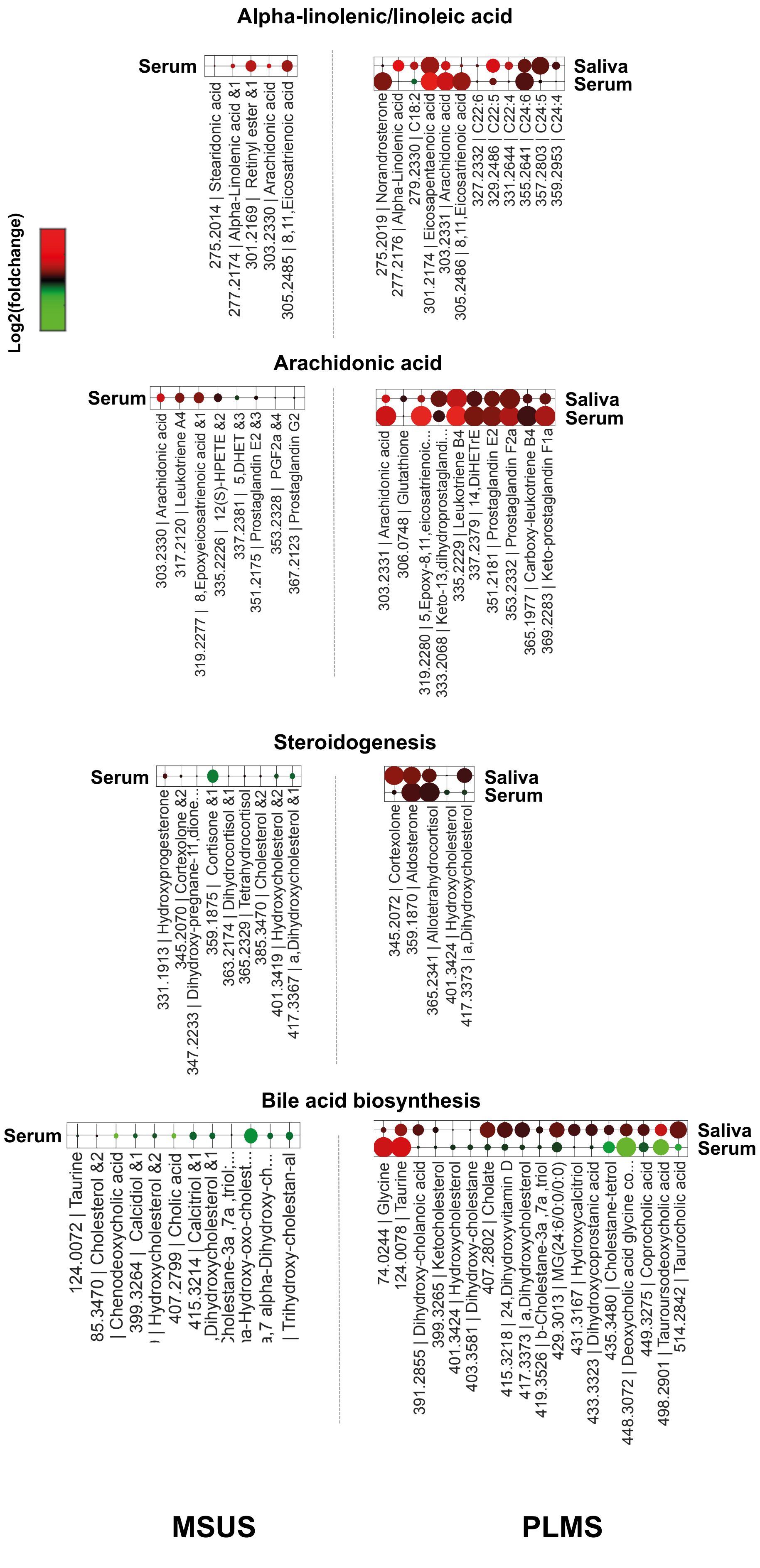

Alpha-linolenic/linoleic acid
Serum
275.2014 | Stearidonic acid
277.2174 | Alpha-Linolenic acid &1
301.2169 | Retinyl ester &1
303.2330 | Arachidonic acid
305.2485 | 8,11,Eicosatrienoic acid
Saliva
Serum
275.2019 | Norandrosterone
277.2176 | Alpha-Linolenic acid
279.2330 | C18:2
301.2174 | Eicosapentaenoic acid
303.2331 | Arachidonic acid
305.2486 | 8,11,Eicosatrienoic acid
327.2332 | C22:6
329.2486 | C22:5
331.2644 | C22:4
355.2641 | C24:6
357.2803 | C24:5
359.2953 | C24:4
Log2(foldchange)
Arachidonic acid
Serum
303.2330 | Arachidonic acid
317.2120 | Leukotriene A4
319.2277 | 8,Epoxyeicosatrienoic acid &1
335.2226 | 12(S)-HPETE &2
337.2381 | 5,DHET &3
351.2175 | Prostaglandin E2 &3
353.2328 | PGF2a &4
367.2123 | Prostaglandin G2
Saliva
Serum
303.2331 | Arachidonic acid
306.0748 | Glutathione
319.2280 | 5,Epoxy-8,11,eicosatrienoic...
333.2068 | Keto-13,dihydroprostaglandi...
335.2229 | Leukotriene B4
337.2379 | 14,DIHETrE
351.2181 | Prostaglandin E2
353.2332 | Prostaglandin F2a
365.1977 | Carboxy-leukotriene B4
369.2283 | Keto-prostaglandin F1a
Steroidogenesis
Serum
331.1913 | Hydroxyprogesterone
345.2070 | Cortexolone &2
347.2233 | Dihydroxy-pregnane-11,dione...
359.1875 | Cortisone &1
363.2174 | Dihydrocortisol &1
365.2329 | Tetrahydrocortisol
385.3470 | Cholesterol &2
401.3419 | Hydroxycholesterol &2
417.3367 | a,Dihydroxycholesterol &1
Saliva
Serum
345.2072 | Cortexolone
359.1870 | Aldosterone
365.2341 | Allotetrahydrocortisol
401.3424 | Hydroxycholesterol
417.3373 | a,Dihydroxycholesterol
Bile acid biosynthesis
Serum
124.0072 | Taurine
85.3470 | Cholesterol &2
| Chenodeoxycholic acid
399.3264 | Calcidiol &1
) | Hydroxycholesterol &2
407.2799 | Cholic acid
415.3214 | Calcitriol &1
Dihydroxycholesterol &1
;holestane-3a ,7a ,triol;...
ıa-Hydroxy-oxo-cholest...
a,7 alpha-Dihydroxy-ch...
| Trihydroxy-cholestan-al
Saliva
Serum
74.0244 | Glycine
124.0078 | Taurine
391.2855 | Dihydroxy-cholanoic acid
399.3265 | Ketocholesterol
401.3424 | Hydroxycholesterol
403.3581 | Dihydroxy-cholestane
407.2802 | Cholate
415.3218 | 24,Dihydroxyvitamin D
417.3373 | a,Dihydroxycholesterol
419.3526 | b-Cholestane-3a ,7a ,triol
429.3013 | MG(24:6/0:0/0:0)
431.3167 | Hydroxycalcitriol
433.3323 | Dihydroxycoprostanic acid
435.3480 | Cholestane-tetrol
448.3072 | Deoxycholic acid glycine co...
449.3275 | Coprocholic acid
498.2901 | Tauroursodeoxycholic acid
514.2842 | Taurocholic acid
MSUS
PLMS

Appendix Figure S6

Individual metabolites annotated to each of the significant enrichments for MSUS adult plasma and PLMS saliva and serum. Colour in circles represents fold change and size represents -log10(adjusted p-value). The largest circle symbolizes adjusted $P < 0.0001$. Metabolites are listed by ion mass followed by their official name. HPETE; 5-hydroperoxyeicosatetraenoic acid. DHET; dihydroxyeicosatrienoic acid. PGF2a; prostaglandin F2a. DiHETErE; dihydroxyeicosatrienoic acid. HETE; hydroxyeicosatetraenoic acid.

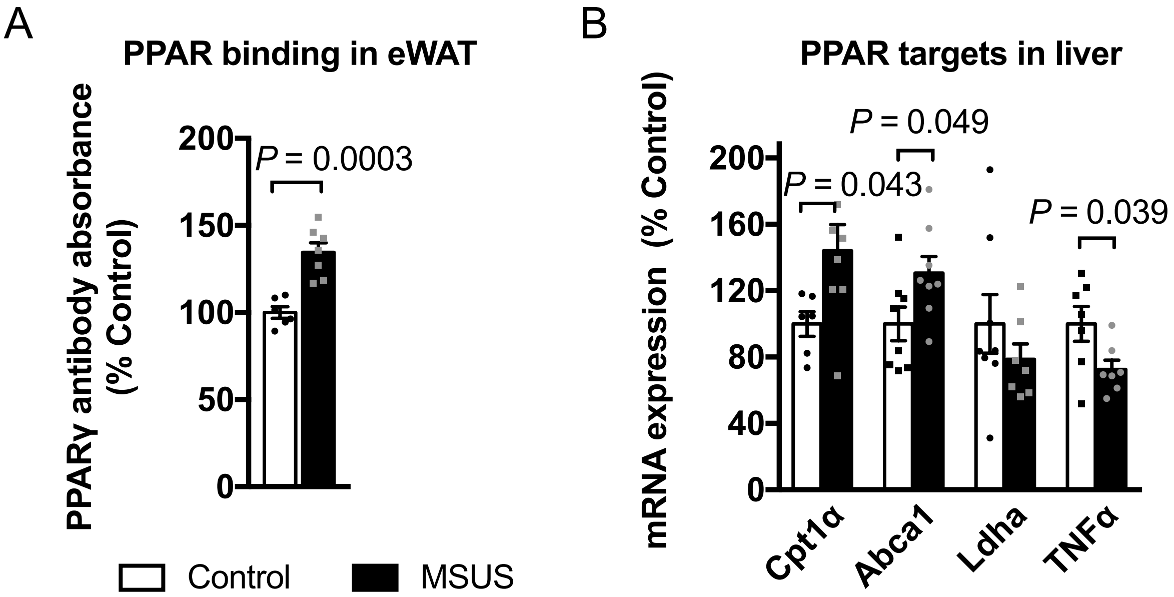

Appendix Figure S7

(A) PPARγ transcription factor binding on consensus sequence using nuclear extracts from epididymal white adipose tissue (eWAT) collected from MSUS and control males. MSUS n = 7, Control n = 6, two-tailed Student's t-test, P = 0.0003, t = 5.23, df = 11. (B) Gene expression analysis of PPAR targets in F1 liver. Cpt1α MSUS n = 8, Control n = 6, P = 0.04, t = 2.26, df = 12; Abca1 n = 8 per group, P = 0.04, t = 2.16, df = 14; Ldhα MSUS n = 7, Control n = 8, P = 0.33, t = 1.02, df = 13; Tnfα n = 7 per group, P = 0.03, t = 2.31, df = 12. Data reported as mean ± s.e.m., for all analyses two-tailed Student's t-test was used.

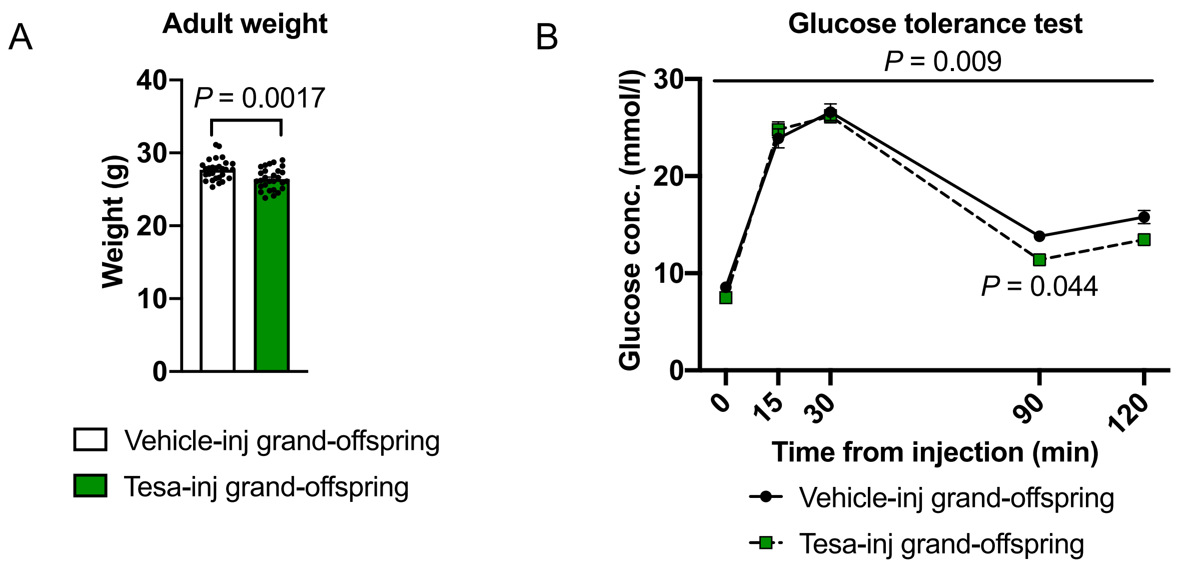

Appendix Figure S8

(A) Weight of grand-offspring of tesaglitazar-injected (Tesa-inj, *n* = 28) and vehicle-injected (Vehicle-inj, *n* = 28) males. Two-tailed Student's *t*-test, *P* = 0.0017, *t* = 3.31, df = 54. Data reported as mean ± s.e.m. **(B)** Glucose level during a glucose tolerance test in the grand-offspring of Tesa-inj compared to Vehicle-inj, *n* = 16 per group, repeat measures ANOVA, treatment effect *P* = 0.106, *F* (1, 28) = 2.796, time effect *P* < 0.0001, *F* (4, 112) = 458.1, interaction *P* = 0.009, *F* (4, 112) = 3.573. conc.; concentration. Data reported as mean ± s.e.m.

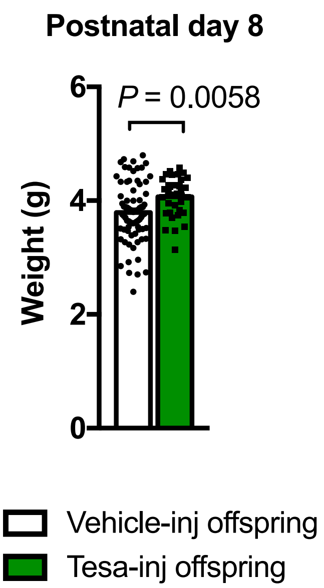

Appendix Figure S9

Weight of offspring (pooled males and females) of tesaglitazar-injected (Tesa-inj, n = 34) and vehicle-injected (Vehicle-inj, n = 78) males at PND8. Two-tailed Student's t-test, P = 0.0058, t = 2.81, df = 110. Data reported as mean ± s.e.m.

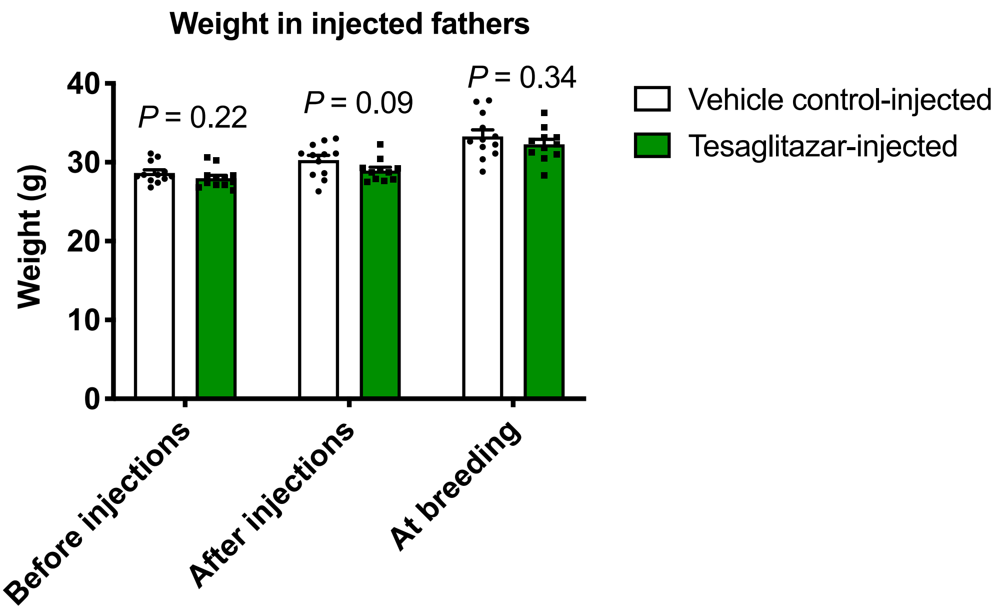

Appendix Figure S10

No change in weight was observed in males injected with tesaglitazar, compared to vehicle-injected males. Measurements were taken before and after injections, then at the time of breeding. For all measurements n = 11. Before injections, Student's *t*-test, *P* = 0.219, *t* = 1.27, df = 22; After injections, Student's *t*-test, *P* = 0.094, *t* = 1.75, df = 21; At breeding, Student's *t*-test, *P* = 0.339, *t* = 0.98, df = 21.

A

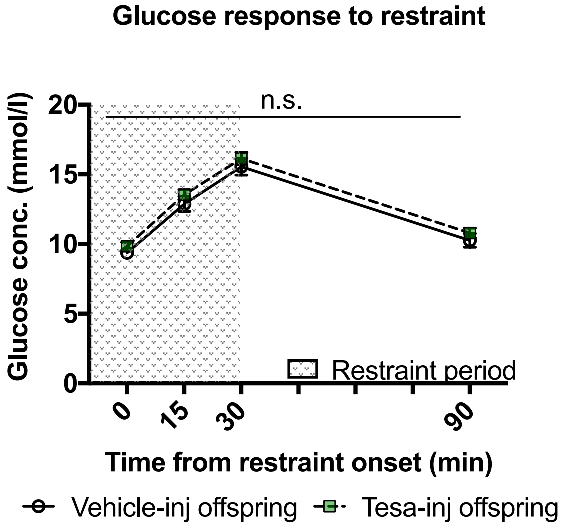

B

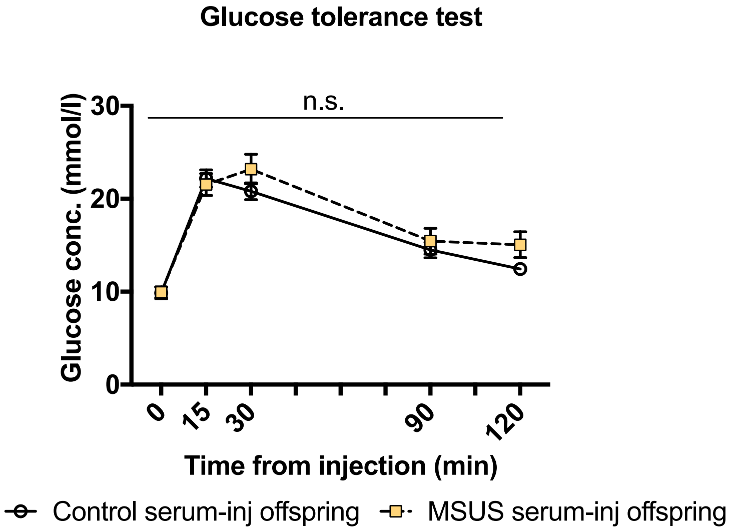

Appendix Figure S11

(A) Glucose level during and after a 30-min restraint challenge in male offspring from tesaglitazar-injected (Tesa-inj, *n* = 17) males compared to vehicle-injected (Vehicle-inj, *n* = 16) males. Repeated measures ANOVA, treatment effect *P* = 0.174, *F* (1, 30) = 1.94, time effect *P* < 0.0001, *F* (3, 90) = 1.941, interaction *P* = 0.99, *F* (3, 90) = 0.024. (B) Glucose level during a glucose tolerance test in the offspring of MSUS serum-injected males compared to controls, *n* = 16 per group, repeat measures ANOVA, treatment effect *P* = 0.109, *F* (1, 150) = 2.598, time effect *P* < 0.0001, *F* (4, 150) = 50.75, interaction *P* = 0.46, *F* (4, 150) = 0.914. n.s.; not significant, conc.; concentration. Data reported as mean ± s.e.m.

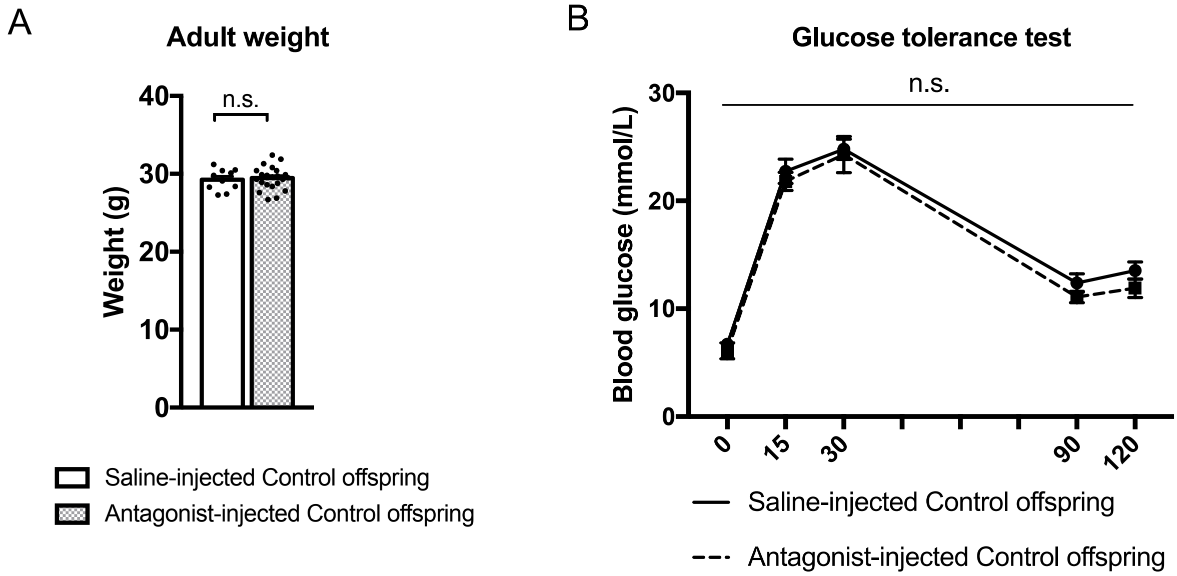

Appendix Figure S12

(A) Adult weight in the offspring of males injected the PPAR antagonist T0070907 (*n* = 22) or saline (*n* = 10). Student's *t*-test, *P* = 0.713, *t* =0.365, df = 30. (B) Blood glucose levels during a glucose tolerance test in saline-injected control offspring (*n* = 8) and antagonist-injected control offspring (*n* = 7). Repeated measures ANOVA, treatment effect *P* = 0.19, F (1, 13) = 1.93, time effect *P* < 0.0001, F (4, 52) = 158.3, interaction *P* = 0.96, F (4, 52) = 0.154. n.s.; not significant. Data reported as mean ± s.e.m.

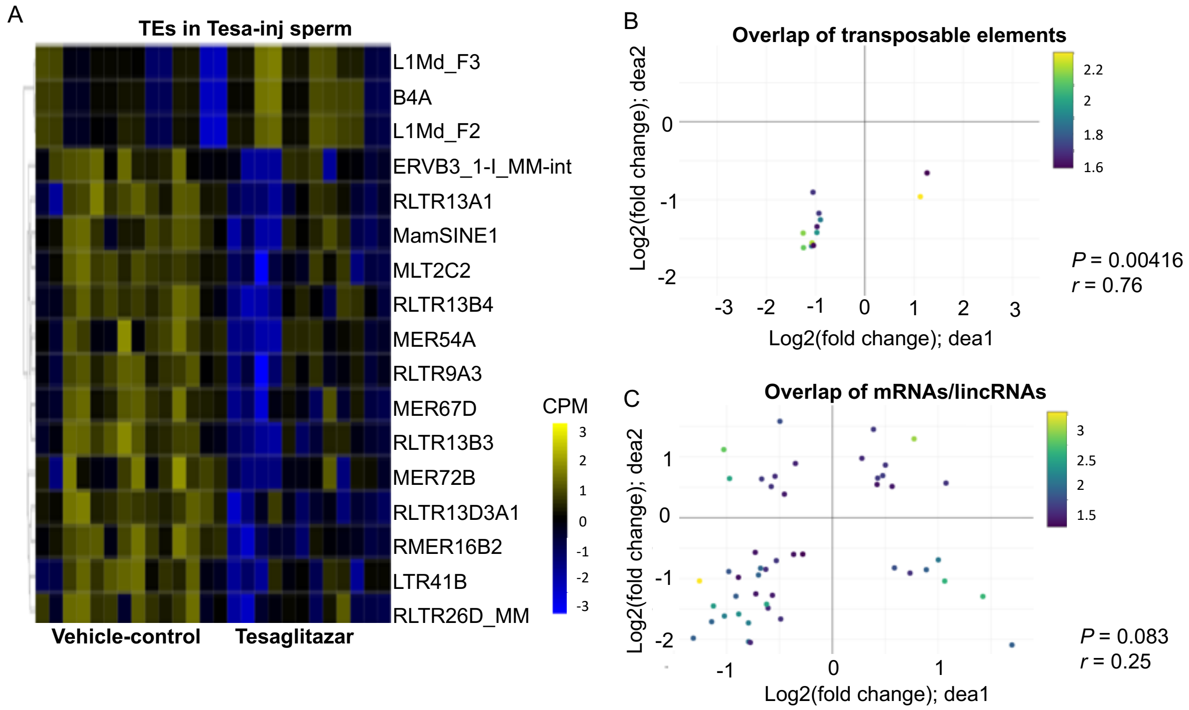

Appendix Figure S13

(A) Most significant transposable elements in sperm from tesaglitazar-injected males (Tesa-inj males, $P < 0.05$). For Tesa-inj n = 6 (duplicate measurements shown); for Vehicle control-injected n = 7 (duplicate measurements shown). Overlap of differentially expressed (B) transposable elements and (C) mRNAs/lincRNAs in sperm from Tesa-inj and MSUS males. A *P*-value cut-off of $P < 0.05$ for both data sets was used for the analysis. The *P*-value and Pearson correlation (*r*) between data sets are indicated on the figure next to the graph. The x-axis represents fold change in tesaglitazar-injected males (dea1), the y-axis represents fold change in MSUS males (dea2). Color legend represents log10(*P*-value) for each individual gene. TEs; transposable elements.

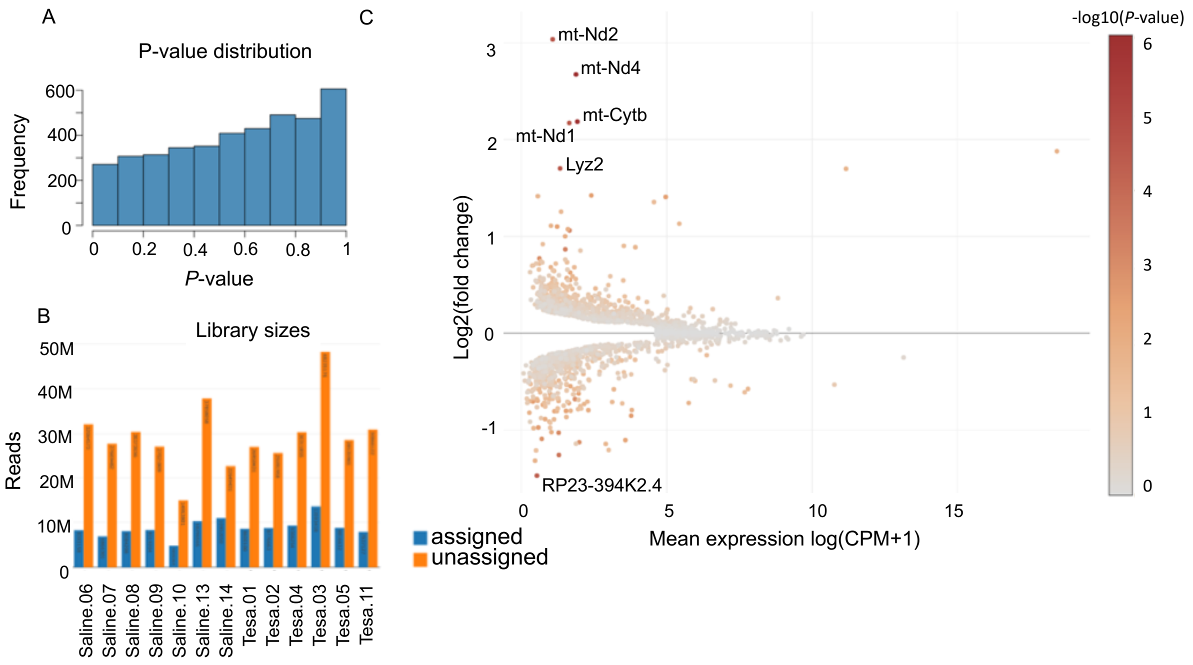

Appendix Figure S14

Descriptive data of sperm RNA sequencing for tesaglitazar- and vehicle-injected males. (A) *P*-value distribution and (B) proportion of assigned and unassigned reads from samples used for analysis. (C) MA plot of differentially expressed mRNA/lincRNAs in sperm from tesaglitazar-injected males. The genes shown have a significance level of FDR < 0.05. $n = 7$ per group.

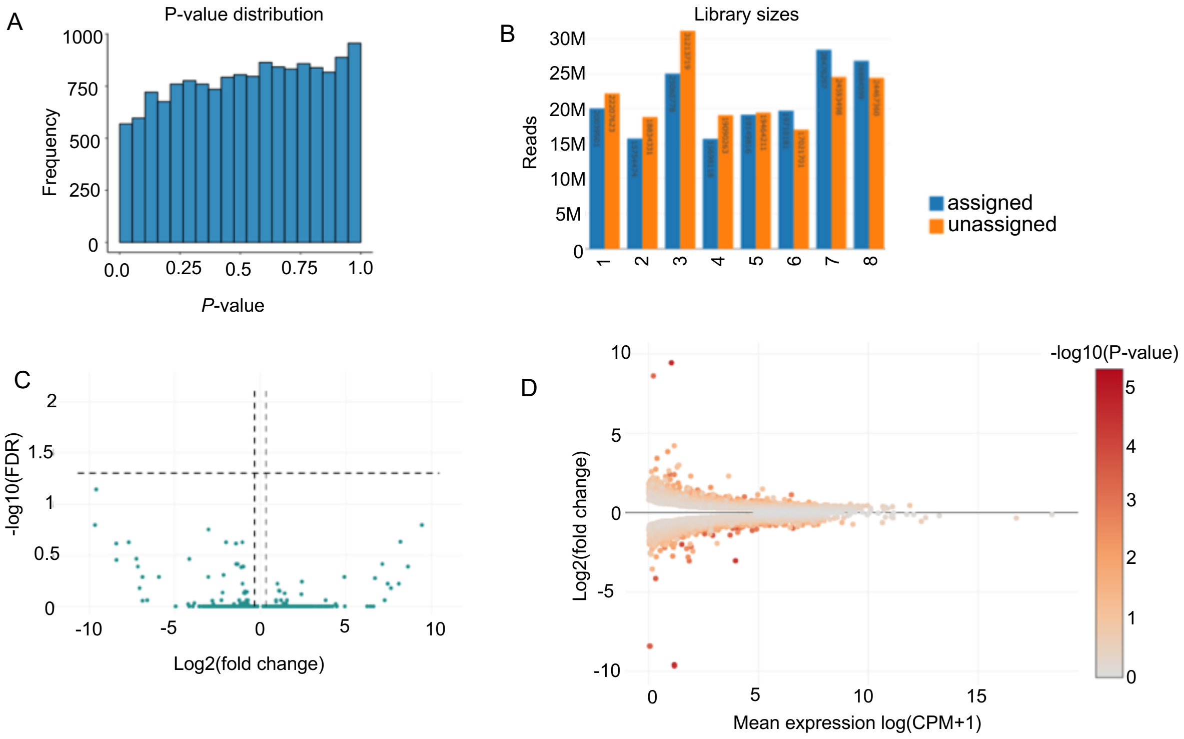

Appendix Figure S15

Descriptive data of sperm RNA sequencing for tesaglitazar- and vehicle-injected males 1-day after the last injection. (A) *P*-value distribution and (B) proportion of assigned and unassigned reads from samples used for analysis. (C) Volcano plot and (D) MA plot (log ratio and mean average) of differentially expressed (FDR < 0.05) mRNA/lincRNAs in sperm from tesaglitazar-injected males (none significant). The genes shown have a significance level of FDR < 0.05. *n* = 7 per group.

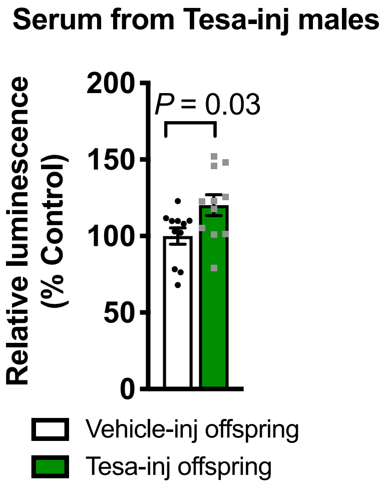

Appendix Figure S16

Relative luciferase luminescence in GC-1 cells transfected with PPRE plasmid and exposed to serum collected from males 24-hours after a single injection of tesaglitazar. Tesa-inj n = 11, Vehicle control n = 11, two-tailed Student's t-test, P = 0.030, t = 2.336, df = 20.

Metabolite name	log2(FC)	p-value	Adjusted p-value (BH)
PC(15:0/P-18:1(11Z))	-0.3229	0.000928	0.160999079
Bilirubin glucuronide	0.285193	0.001032	0.160999079
Cholesterol sulfate	-0.32606	0.00231	0.240274042
PE(24:0/P-18:1(11Z))	-0.21815	0.004723	0.368431177
PE(18:4(6Z,9Z,12Z,15Z)/P-16:0)	0.359331	0.006303	0.393329688
Aminoadipate	-0.26152	0.007915	0.411587649
CE(20:5(5Z,8Z,11Z,14Z,17Z)	0.459773	0.014317	0.453611838
PE(18:1(11Z)/P-18:1(11Z))	-0.21179	0.01754	0.453611838
3-O-Sulfogalactosylceramide (d18:1/24:0)	-0.10851	0.017618	0.453611838
PE(22:0/P-18:0)	-0.25795	0.017683	0.453611838
Hexonic acid	-0.21292	0.018594	0.453611838
Oxidized glutathione	-0.21925	0.018681	0.453611838
Leukotriene A4	0.217496	0.0189	0.453611838
PE(22:2(13Z,16Z)/22:6(4Z,7Z,10Z,13Z, 16Z,19Z))	-0.20292	0.021091	0.47002934
Vitamin A	0.375696	0.023447	0.487690975
3-O-Sulfogalactosylceramide (d18:1/20:0)	-0.14853	0.025282	0.493005589
Itaconate	-0.1586	0.029515	0.513155302
PE(22:4(7Z,10Z,13Z,16Z)/P-18:1(11Z))	-0.16405	0.029605	0.513155302
SM(d18:0/16:1(9Z))	-0.18857	0.032129	0.527586127
Bilirubin	0.642463	0.034612	0.537670051
Stearidonic acid	-0.28706	0.038088	0.537670051
PC(24:0/P-18:1(11Z))	-0.10549	0.038108	0.537670051
Hexuronate	-0.17405	0.040281	0.537670051
PC(15:0/24:0)	-0.07857	0.041359	0.537670051
beta-Alanyl-L-lysine	-0.26194	0.049541	0.578169611

Appendix Figure S17

Metabolites identified in plasma from tesaglitazar-injected (*n* = 5) and vehicle-injected (*n* = 5*)* males at breeding 46 days after the last injection. *P*-values were corrected using Benjamini-Hochberg (BH) post-hoc test.

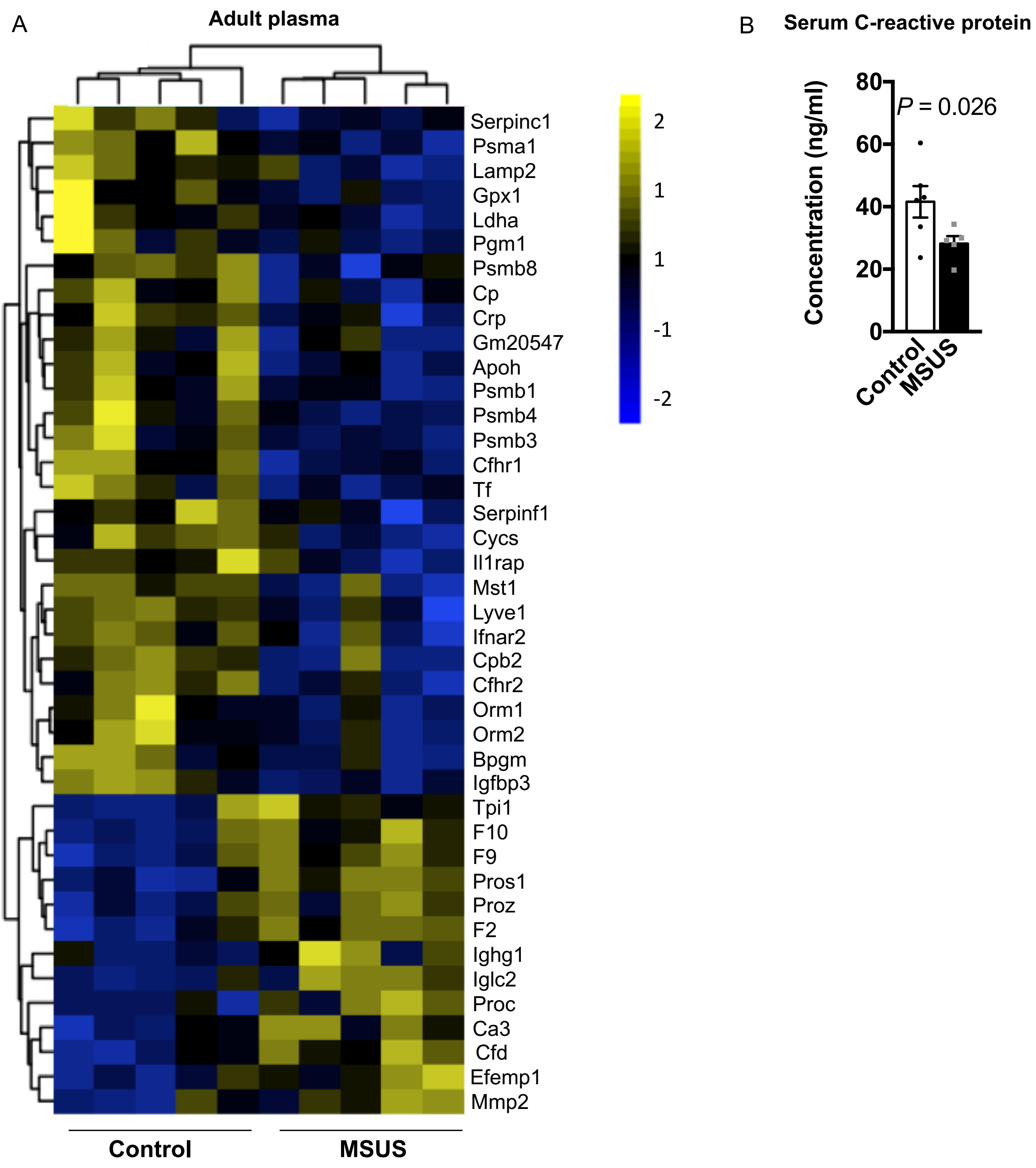

Appendix Figure S18

(A) Heat map of differentially expressed proteins in plasma from F1 MSUS and control males. All shown proteins have $P < 0.05$ after ANOVA but do not pass multiple testing corrections (FDR > 0.05), $n = 5$ per group. CRP (underlined) is validated using independent samples in panel b. (B) Serum CRP in MSUS ($n = 5$) and control ($n = 6$) males. One-tailed Student's t-test, $P = 0.026$, $t = 2.23$ df = 9. Data reported as mean ± s.e.m.

name	logCPM	p-value	FDR
mmu-mir-221-3p	7.78473061	0.00892147	0.84753953
mmu-mir-19a-3p	3.96192968	0.02416109	1
mmu-mir-19b-3p	4.92982496	0.11563418	1

Appendix Figure S19

Differential expression analyses of small RNA sequencing in mouse serum. Table shows RNAs with $P < 1$.

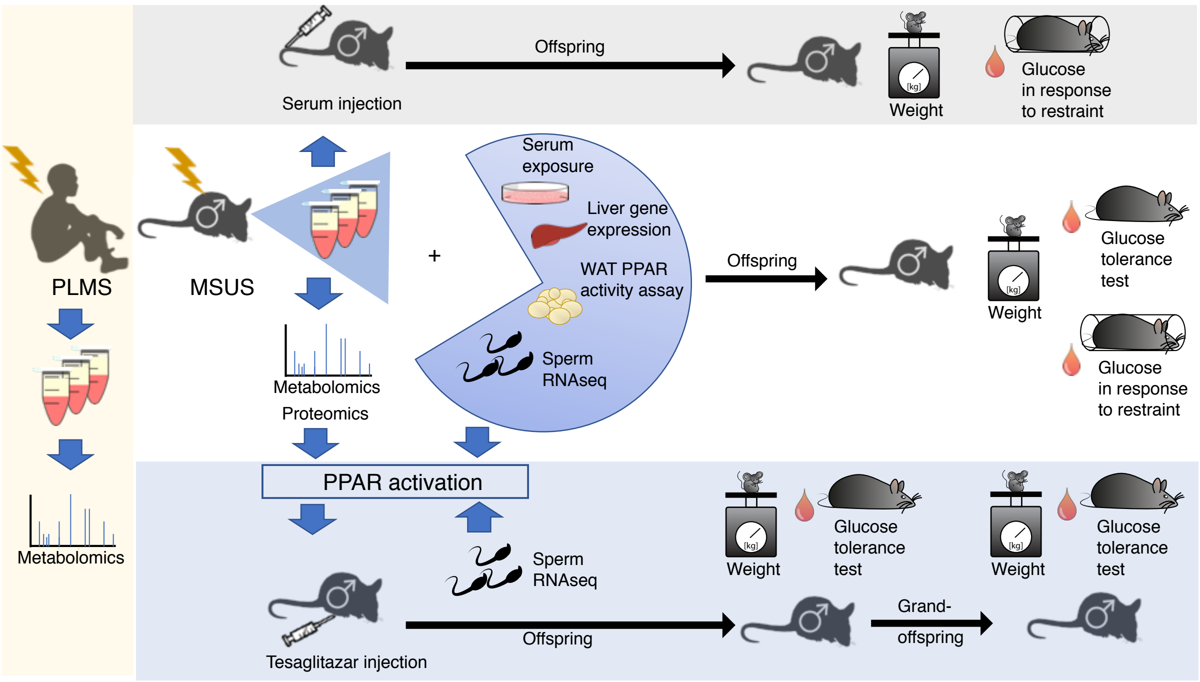

Appendix Figure S20

Schematic illustration of workflow and summary of experiments.

	Fathers		Offspring	
**Experiment**	**Vehicle-injected**	**Tesaglitazar-injected**	**Vehicle-injected offspring**	**Tesaglitazar-injected offspring**
Pup weight	11	5	78 (37 males)	34 (17 males)
Adult weight	9	5	24	17
GTT	10	5	26	16
Glucose Restraint	7	5	17	16

	Fathers		Offspring	
**Experiment**	**Control**	**MSUS**	**Control offspring**	**MSUS offspring**
Adult weight	7	12	24	22
Glucose Restraint	6	9	15	16

	Fathers		Offspring	
**Experiment**	**Control serum-injected**	**MSUS serum-injected**	**Control serum-injected offspring**	**MSUS serum-injected offspring**
Adult weight	7	8	32	32
GTT	4	5	8	8
Glucose Restraint	4	5	8	8

Appendix Figure S21

Number of fathers and offspring for each experiment. Numbers are reported after outlier removal due to illness, death or performance deviating from the norm (2StDev).

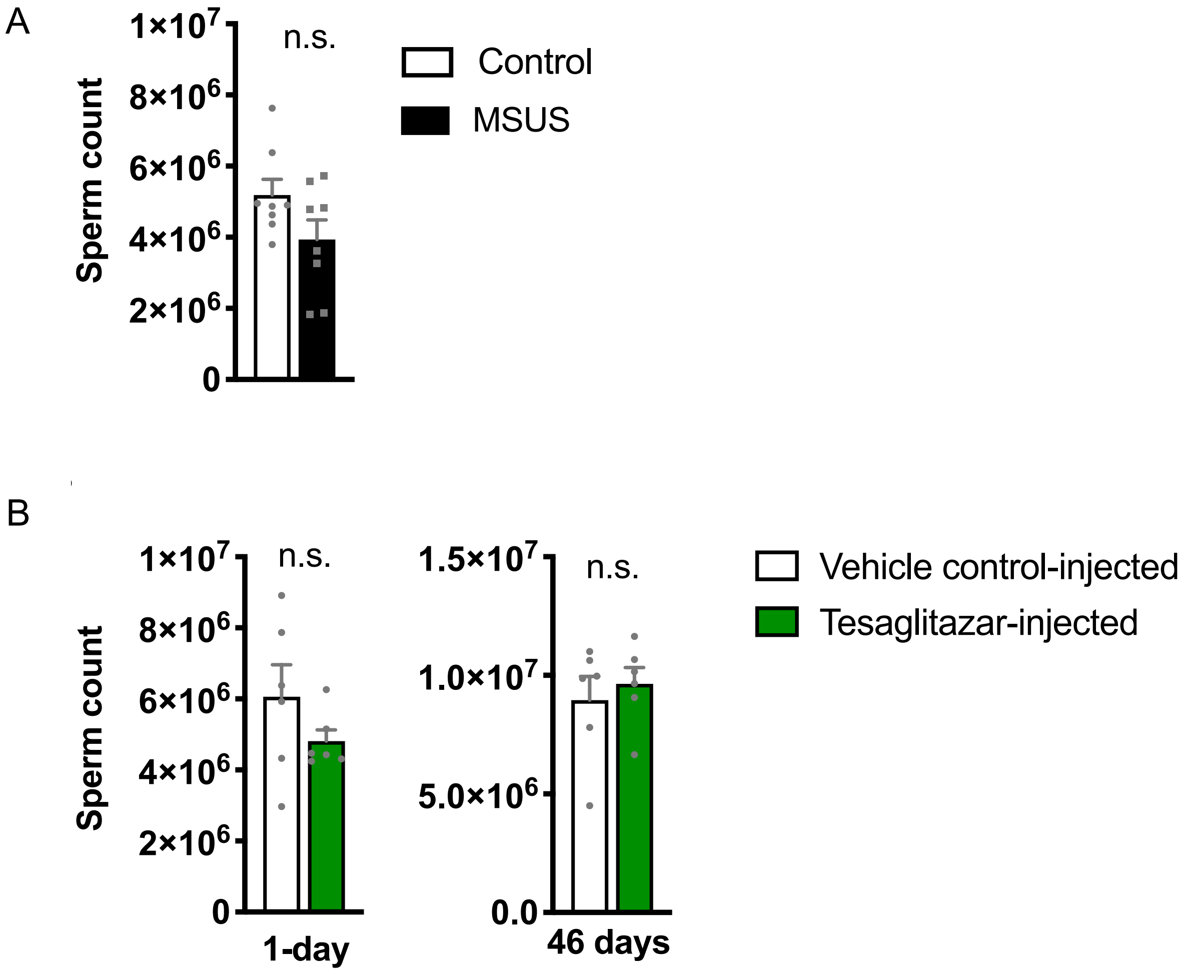

Appendix Figure S22

No difference in sperm count in MSUS males or in males treated with tesaglitazar. Number of sperm cells in (A) Control (n = 8) and MSUS (n = 8) males, 2-tailed Student's t-test, P = 0.094, t = 1.797, df = 14, and in (B) males treated with tesaglitazar, 1 day or 46 days after the final injection. Vehicle control-injected, n = 6, Tesaglitazar-injected, n = 6. 1 day after: 2-tailed Mann-Whitney test, P = 0.31, Mann-Whitney U = 11. 46 days after: 2-tailed Student's t-test, P = 0.59, t = 0.555, df = 10.

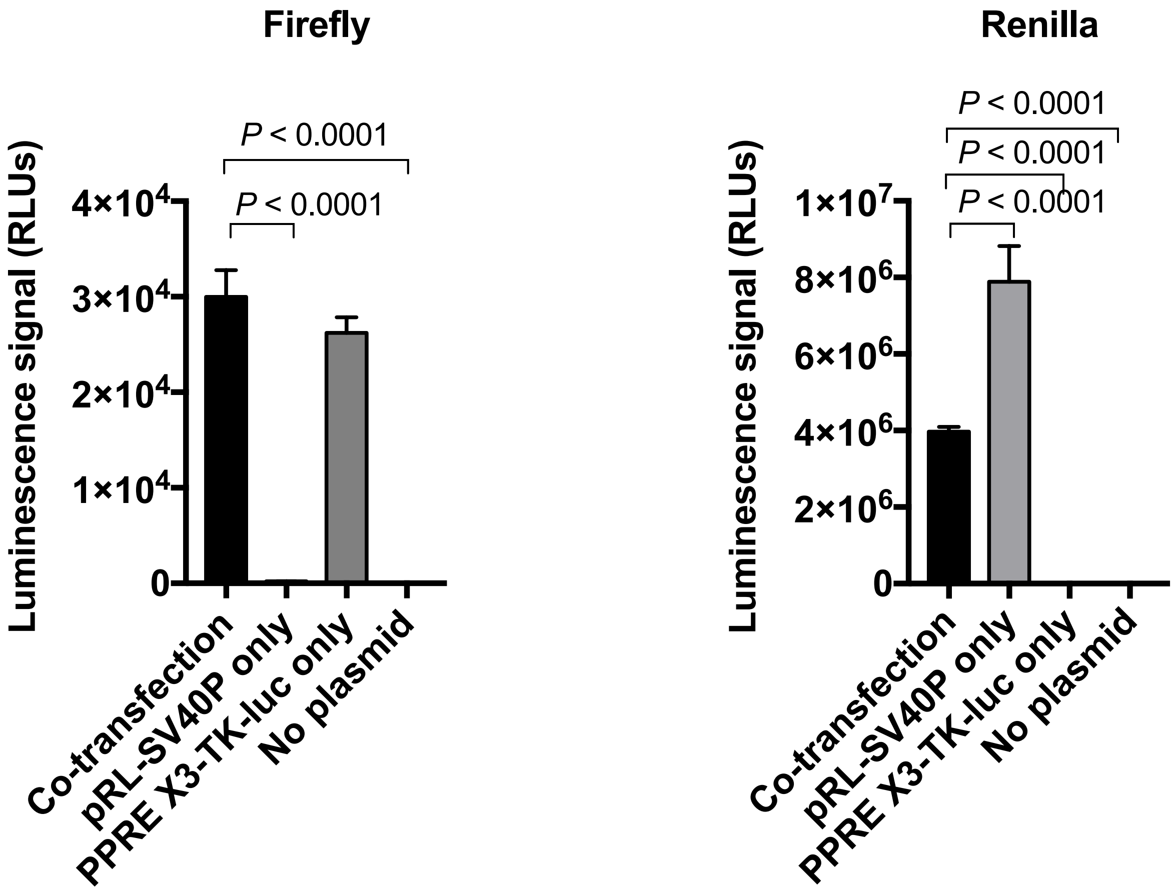

Appendix Figure S23

Baseline renilla and firefly luminescence signals after exposure to control serum in cells co-transfected with both pRL-SV40P and PPRE X3-TK-luc or transfected with each construct alone. pRL-SV40P expresses renilla luciferase used for normalization and PPRE X3-TK-luc is a PPAR response element reporter expressing firefly luciferase upon PPAR binding. Co-transfection (n = 19; Control serum), pRL-SV40P only (n = 8; Control serum), PPRE X3-TK-luc only (n = 8; Control serum), or no plasmid (n = 8; Control serum). One-way ANOVA (Tukey's multiple comparison corrections), for Firefly $P < 0.0001$, $F(3, 39) = 38.41$, for Renilla $P < 0.0001$, $F(3, 39) = 83.5$.
